# In Anticipation of Sharing Pediatric Inpatient Notes: Focus Group Study With Stakeholders

**DOI:** 10.2196/37759

**Published:** 2022-05-30

**Authors:** Catherine Arnott Smith, Michelle M Kelly

**Affiliations:** 1 The Information School University of Wisconsin-Madison Madison, WI United States; 2 Department of Pediatrics School of Medicine and Public Health University of Wisconsin Madison, WI United States

**Keywords:** medical informatics, information sharing, electronic health records, patient portals, hospitals, pediatrics, focus group, inpatient care, caregivers

## Abstract

**Background:**

Patient portals are a health information technology that allows patients and their proxies, such as caregivers and family members, to access designated portions of their electronic health record using mobile devices and web browsers. The Open Notes initiative in the United States, which became federal law in April 2021, has redrawn and expanded the boundaries of medical records. Only a few studies have focused on sharing notes with parents or caregivers of pediatric patients.

**Objective:**

This study aimed to investigate the anticipated impact of increasing the flow of electronic health record information, specifically physicians’ daily inpatient progress notes, via a patient portal to parents during their child’s acute hospital stay—an understudied population and an understudied setting.

**Methods:**

A total of 5 in-person focus groups were conducted with 34 stakeholders most likely impacted by sharing of physicians’ inpatient notes with parents of hospitalized children: hospital administrators, hospitalist physicians, interns and resident physicians, nurses, and the parents themselves.

**Results:**

Distinct themes identified as benefits of pediatric inpatient Open Notes for parents emerged from all the 5 focus groups. These themes were communication, recapitulation and reinforcement, education, stress reduction, quality control, and improving family-provider relationships. Challenges identified included burden on provider, medical jargon, communication, sensitive content, and decreasing trust.

**Conclusions:**

Providing patients and, in the case of pediatrics, caregivers with access to medical records via patient portals increases the flow of information and, in turn, their ability to participate in the discourse of their care. Parents in this study demonstrated not only that they act as monitors and guardians of their children’s health but also that they are observers of the clinical processes taking place in the hospital and at their child’s bedside. This includes the clinical documentation process, from the creation of notes to the reading and sharing of the notes. Parents acknowledge not only the importance of notes in the clinicians’ workflow but also their collaboration with providers as part of the health care team.

## Introduction

### Background

In 2001, the Institute of Medicine acknowledged that the “free flow of information” between patients and electronic health record (EHR) systems is central to the principle of patient-centered care [1]. Tang and Lansky [2] proposed that patients need access to their personal health information, at minimum “their own diagnoses, medications, allergies, lab test results, visit summaries, and other findings over time.” They further argued that access to this information could enable patients to enter into a true dialogue with their health care team, increasing not only their understanding of the treatment required but their motivation to engage in such treatment—essentially establishing themselves as the locus of control in the relationship.

Patient portals are a health information technology that allows patients and their proxies, such as caregivers and family members, to access designated portions of their EHR using mobile devices and web browsers [3]. Patients are now potential readers and users of EHRs. This has redrawn and expanded the boundaries of medical records. In this way, patient portal technology has been deemed a “digital disruption” in the health care industry—a “type of environmental turbulence induced by digital innovation that leads to the erosion of boundaries and approaches that previously served as foundations for organizing the production and capture of value” [4]. Sullivan and Staib [5] reported that over 50% of EHR implementations fail because organizations do not appreciate the degree to which such digital transformations can be disruptive. These authors further identify various “syndromes” associated with digital disruptions, including digital deceleration (reduced efficiency) and hypervigilance (anxiety and overreaction in the face of change) [5].

To facilitate the success of digital transformations in health care and mitigate disruption, a mutual understanding of health information exchange and relevant technologies is required by all stakeholders involved—patients and their caregivers and health care professionals. The objective of this study was to investigate the anticipated impact of increasing the flow of EHR information, specifically physicians’ daily inpatient progress notes, via a patient portal to parents during their child’s acute hospital stay—an understudied population and an understudied setting.

### The Open Notes and Copying Letters Initiatives

Patient access to personal health information in the United States was greatly accelerated in 2010 by a patient-centered movement called Open Notes. The collaborative experiment that launched the movement involved Beth Israel Deaconess in Massachusetts, the Geisinger Clinic in Pennsylvania, and the Harborview Medical Center in Washington State. At these 3 centers, 20,000 patients were invited to read their ambulatory visit notes written by their clinicians using their patient portals. Results were overwhelmingly positive, with 59% to 77% of patient survey respondents agreeing that viewing their clinicians’ notes helped them feel “more in control of their care” [6]. Since then, 51% of US adults who accessed their medical records via web in 2020 reported that these records included clinical notes [7].

The Copying Letters initiative, which began in the United Kingdom in the 2000s, presents an interesting and relevant initiative parallel to Open Notes. Launched in April 2004, this initiative was similarly grounded on the idea that all patients should carry a summary of their medical record [8]. To enable this summary to be as complete as possible, all clinicians were required by the National Health Service to send their patients copies of all letters they had written about them, for example, a letter describing their case in the context of a referral to another specialist [9]. The cited benefits of this practice were very similar to those articulated in the Open Notes movement. Supporters argued that, by providing access to the contents of the record, Copying Letters put the patient “at the centre of care” [10] and effects “a shift in the balance of power” [11].

Advocates of medical record transparency argue that there are many additional benefits, including enhanced physician-patient communication, improvements in patient understanding of their own condition and ability to perform self-care, and increased patient engagement and participation [12,13]. Therefore, this transparency has now been mandated by law in the United States under the 21st Century Cures Act. Effective April 2021, a total of 8 types of clinical notes—consultation, discharge summaries, history and physicals, imaging, laboratory reports, pathology reports, procedure, and progress notes—must be shared with patients [14].

### Pediatric Inpatient Context

Although the Open Notes and Copying Letters initiatives have both been adopted at a national level, only a few studies have focused on sharing notes with parents or caregivers of pediatric patients. Early studies of Copying Letters were conducted because some pediatricians were concerned about the effect of sharing clinical content on adolescent patients and parent readers [15]. They feared not only that these readers would be confused but that any sensitive information might be stigmatizing or offensive. These studies were built on the early work by Partridge [15] who explored parental reactions after reading their child’s pediatrician’s assessment reports.

The body of Copying Letters research repeatedly documents the ways in which parents, as in-home managers of their children’s health, perceive themselves as silent partners of physicians [15-18]. It is clear that these parents valued access to clinician-authored documentation of their child’s care. A very early study by Partridge [15] found general satisfaction with pediatricians’ letters among parents of children living with disabilities, with 74% of parents being satisfied with what they read. Other researchers found similarly high rates of satisfaction among patient readers. Cowper and Lenton [17] reported: “One hundred percent of parents were pleased to have received the letter” from their child’s pediatrician. Liapi et al [19] compared adult patients in an otolaryngology clinic with 100 parents of pediatric patients; 77% of the parents found the copied letters helpful. Most recently, Amirav et al [18], who surveyed parents of pediatric patients with asthma, reported that 80% of the parents called the letters "helpful" and 98% indicated that they would want similar letters in the future.

In the United States, the Open Notes movement began with adult outpatients. Researchers have only now started to investigate the access behaviors of inpatients and their reactions to content, ranging from medication information [20] to their full medical record [21]. A review of the medical literature reveals a small but growing body of literature on access to medical records by hospitalized patients but finds even less research in pediatrics [22]. This mirrors the general situation for EHRs and personal health records, in which the research literature largely concerns adult outpatients [23,24]. Therefore, there are significant gaps in our understanding of patient- and caregiver-facing tools in the context of inpatient care [25], particularly Open Notes in the pediatric hospital setting. Instead, much of the research published on young patients focuses on the complexities of policies surrounding access to patient portals by children and adolescents [26]. One research group at the Boston Children’s Hospital designed a framework for a system of personally controlled health records to be accessed by “parents, guardians, and third-party entities” while remaining in the patient’s control [27].

Kelly et al [28,29] were the first to investigate the use of an inpatient portal by parents of pediatric patients, increasing our understanding of parental motivations for accessing their child’s inpatient records in real time. Of the 14 parents interviewed in that study, 13 were interested in having access to physicians’ notes in the portal:

I don’t know that doctors necessarily keep it a secret, but in my son’s entire medical history, I’ve only had one doctor really turn the screen to me and sit there and say like “Here’s what we’re seeing, here’s what’s happening.” So, if I could see things like [notes] in here, that would be amazing.

Parent

Parents suggested that notes would provide a recapitulation of information, serve as a memory aid, and improve their understanding and ability to advocate for their child:

Sometimes talking is different than writing. Sometimes I will forget the point. [With notes], we’ll know where’s the problem and what’s the next step.

Parent

When you read, you can understand it much better.

Parent

Others suggested that they would like to refer to notes when they were unavailable during morning rounds:

I wasn’t here [during rounds]. So, if they say the doctor’s notes are on there, I could be able to read them and see what [the doctor’s] suggesting.

Parent

However, some parents had concerns that notes could cause undue anxiety and had reservations about the impact of sharing on physicians:

I don’t know how comfortable [doctors] would feel. It may feel like an invasion of [doctors’] privacy.

Parent

This study builds on these early findings and continues our systematic approach [30] to evaluate the perspectives of key people potentially involved in this digital disruption—the sharing of physicians’ notes with families of hospitalized children. These findings will allow for a mutual understanding of stakeholder perspectives and facilitate the success of note sharing in light of recent federal mandates.

## Methods

### Study Design and Participants

This qualitative study was conducted at a Midwest academic children’s hospital between October and November 2018. A total of 5 in-person focus groups were conducted with 5 different types of stakeholders considered most likely to be impacted by sharing of physicians’ inpatient notes with parents of hospitalized children. There were no exclusion criteria; 4 groups were composed of hospital staff with the roles described later, whereas parents were recruited from the hospital’s Patient and Family Advisory Council, a standing committee of volunteers. Separate focus groups were conducted for each stakeholder role to encourage participants to respond freely, without the fear of retribution. Adolescents were intentionally excluded from the focus groups because of the complexities of access to adolescent health information.

Focus group participants were recruited via email. All participants were provided with an information sheet describing the study and risks and benefits. Informed consent was obtained; participants were not reimbursed.

### Ethics Approval

This study was approved by the institutional review board of the University of Wisconsin-Madison (protocol ID number: 2018-0913).

### Focus Groups

Each group met in a private conference room for 1 session; the sessions lasted 1.5 to 2 hours and were audio recorded. Using a semistructured facilitator guide consisting of open-ended questions, moderators asked all focus group members for their opinions about the potential of providing parents of patients aged ≤12 years with real-time access to daily inpatient progress notes using a bedside tablet during their child’s hospitalization. To facilitate the discussion, moderators provided an example of a daily progress note and reviewed the general content of these notes with all focus group participants. Participants were then asked to reflect on sharing progress notes with parents.

### Data Analysis

Audio recordings of focus group sessions were transcribed by a professional service, and transcripts were deidentified and coded using Dedoose (version 8.3.17, SocioCultural Research Consultants, LLC). Three researchers participated in coding using a constant comparative method [31,32]. Two researchers (MMK and CAS) independently reviewed all transcripts and met with the third researcher to develop a codebook. These 2 researchers then coded all transcripts and consulted with the third researcher to reach consensus concerning any discrepancies, always referring back to the transcripts [33]. The themes were summarized and presented using illustrative quotes. Further details about the study methods are available in a study by Smith et al [30].

## Results

### Demographics

The 5 focus groups comprised 6 *administrators* (leaders in the hospital and residency program, information services, risk management, and patient relations), 7 pediatric attending *hospitalist physicians* (physicians whose primary professional focus is on hospitalized patients), 5 pediatric *intern* and *resident physicians*, 8 bedside *nurses*, and 8 *parents* who had experience caring for a child in the hospital. A total of 34 participants were included in the 5 groups. These participants were largely White (30/34, 88%) and female (27/34, 79%) and held college degrees (23/34, 68%).

### Benefits of Inpatient Open Notes

Distinct themes identified as benefits of pediatric inpatient Open Notes for parents emerged from all the 5 focus groups. These themes were communication, recapitulation and reinforcement, education, stress reduction, quality control, and improving family-provider relationships.

#### Communication

The value of Open Notes for improving communication between the inpatient health care team and members of the patient’s family was commented on by various focus group participants, but particularly by residents. They also saw notes as a way to improve communication between parent caregivers:

The one potential benefit that I can think of is that in these families that have, say, four children, and one of them is in the hospital, so both parents can’t always be there,...Dad or Mom, if they have to stay home that day, can read the note from that day...

Resident

Communication between members of the health care team and the patient’s family could potentially be enhanced. Multiple participants saw value in making the treatment plan accessible before rounds to increase families’ understanding and potentially change the family dynamic or discussion with the team:

[M]ost of the questions you get overnight are related to the plan...What are we doing?...What’s going to happen tomorrow? [W]hat are we waiting for? [T]he plan is something that may benefit [them], and their having it may reduce the questions.

Resident

One parent who had been able to view her child’s physician notes during a hospital stay at another institution pointed out that seeing the notes gave her information about communication, which was another benefit:

I’ve had the good fortune to see some of the doctor’s notes...it allows you to learn a little bit more about what’s going on...Did so-and-so understand, or did I understand what was being said?

Parent

#### Recapitulation and Reinforcement

Nurses pointed out the usefulness of Open Notes as a tool to empower families with information, relieving parents of the need to ask hospital staff clarifying questions:

[F]amilies know too that we, as nurses, are busy and physicians are busy...later in the day, they could be talking to Dad or another family member, and it just gives them a tool...to be able to speak to and look back without having to necessarily bother us. Because, a lot of times, that’s what they say. “Oh, well, we didn’t want to bother you.”...[I]t would give them an extra tool to look back...

Nurse

Parents valued the idea of Open Notes for providing families with a text-based source of information that reiterated and reinforced what had already been relayed verbally. This was important for recapturing knowledge in the short term:

[P]art of the objective would be to talk about goals and getting released from the hospital, things like that. Sometimes those are multistep, and there’s a lot there, and it’s hard to remember just from a verbal conversation.

Parent

It was also valuable to access this information over time:

[H]aving that at your fingertips is, it’s so much easier when you have to...remember down the line something for the school, or something for a social worker...that you could quick go back and look at...where was he on this scale when he did his neuro test?

Parent

#### Education

A hospitalist commented on the potential value of notes for families as an educational intervention, deployable for people in different learning situations:

[F]amilies should probably end up having better understanding, better health literacy as a result of this, because they will have the words that they missed when someone was talking too fast or...in an accent, or using words they’ve not heard before, that they can now look up at their leisure without feeling embarrassed about asking questions that they weren’t sure they should ask.

Hospitalist

#### Stress Reduction

A mother described how a visible plan would provide her with structure to reduce her anxiety:

For me, my biggest issue with my mental state and my anxiety around my daughter is when something is going on and there’s no plan. I feel like I’m trying to reach someone, you know...trying to get in, trying to be seen, and like there’s that question mark. I don’t know if it’s serious. I don’t know if it’s not serious...I...automatically feel more at ease as a parent when I know that there’s steps that we’re going through to improve the situation. Like there’s a roadmap.

Parent

A nurse voiced her opinion that showing families the breadth and depth of information being collected about the patient would itself serve to lower parental stress:

I think we could eliminate some of the anxiety of the parents just reading that...explaining, we don’t think it’s this, but we are going to rule out this, this, this to make sure that we’re covering all of our bases.

Nurse

#### Quality Improvement

Unsurprisingly, hospital administrators talked most about Open Notes’ potential for improving the quality of health care delivery:

...I think what [Open Notes] will also do is prompt further discussion...if there’s information in the medical record...that a family doesn’t understand or that we’ve written incorrectly, that’s in the medical record now. And so...it’s almost like another set of eyes on what we’re thinking about the path for either the patient or, in this case, our parents.

Administrator

However, both hospitalists and parents also commented on the potential for Open Notes to be used as a mechanism for quality control, describing the potential value of parents in improving the accuracy of medical records:

Hopefully, [parents will] feel like they know the plan better. Maybe they’re going to check, they’re going to see that something is inaccurate.

Hospitalist

[T]here might be critical pieces of information that may not have been stressed enough or could be missed in that period of rounds, and so it gives you the opportunity to say, hey, this other topic...that was really important to me. And only with a second set of eyes would you be able to capture that information...I think it’s really important.

Parent

#### Improving Family-Provider Relationships

Hospital administrators argued that Open Notes could play a role in reassuring parents, and one parent agreed:

Sometimes you’re only in the room, so maybe five, ten minutes, but it’s actually a very complicated case...I am in the room a short time, but my note is extensive. That could give [parents] more reassurance that I did, in fact, think about all the stuff that maybe we didn’t talk about...But they’re like, wow, that person really is thinking about my case.

Administrator

Once I have steps in place, we’re going to check this, rule this out, move onto this, I...automatically feel more at ease as a parent when I know that there’s steps that we’re going through to improve the situation.

Parent

### Challenges of Inpatient Open Notes

The focus group participants also pointed to the particular challenges posed by inpatient Open Notes. The 5 dominant themes were burden on provider, medical jargon, communication, sensitive content, and decreasing trust.

#### Burden on Provider

The most frequent challenge of Open Notes was the idea that transparency and access to notes by parents would place an undue burden on hospital providers. Every provider focus group mentioned this theme, particularly the residents:

[R]ight now, we work 16 hours...and now if you’re going to add on top of that having to run to the parents’ bedside to explain our note, that’s going to delay all our other responsibilities. [I]f you are going to add extra documentation...that’s going to be more work that we aren’t necessarily going to have time to do.

Resident

One hospitalist acknowledged adding the patient’s family as a new reader of the note and spoke about the extra work involved in considering an additional audience during the writing process. They anticipated that learning to write notes for parental viewing could be challenging for residents who were still honing their note-writing skills. They used the analogy of a parent teaching a child how to write:

[W]hen you have your child who’s writing something, you ask them to go back and edit themselves...that is an expectation. And if they’re having trouble with that, then you say this is the checklist of things you need to look for. Does every sentence have a capital? Does every sentence have a piece of punctuation on it?...Are all the words spelled correctly? So, in a similar vein, we almost need a checklist for the residents to say,...have you done this, have you done this, is this accurate, before you submit it to me. Because that would also potentially reduce the amount of time I’m going to spend on doing it.

Hospitalist

In addition, one member of the parent group voiced similar concerns, saying:

I can’t fathom physicians needing to tone it into a different format. That just sounds like a lot of work for whoever is putting that into place. How would you do that?

Parent

Parent participants speculated about the potential impacts of Open Notes on their child’s health care providers. A persistent theme among parents was questioning of the rationale for the Open Notes initiative in general, as opposed to the specific implementation of Open Notes at this hospital or in the pediatric setting. One parent stated bluntly of Open Notes: “I’d be shocked if the doctors really wanted it” (Parent).

Another parent speculated about possible motivations for the Open Notes initiative:

...If physicians are wanting this...is it because they are hoping that parents become more involved?

Parent

One parent reflected on her role as a witness of different specialists consulting at her child’s bedside and referred to the importance of clinical documentation by all these physicians working in partnership:

[It’s] sometimes hard to get the different specialists in the room. They play, in my experience, they play with their brains by writing notes back to each other, or they read each other’s notes.

Parent

Other parents expressed fear that increased transparency of clinical notes would suppress or hinder clinical thinking and dialogue between physicians. These parents were resistant to that potential change:

[D]octors have got to be able to have notes that they can communicate freely so that they can figure out what’s going on with some of these kids, because a lot of times they don’t know...

Parent

I think it’s really important if we’re going to do this that we don’t stifle the care and stifle the doctors from doing their jobs.

Parent

I want doctors to always have the freedom with each other to say, “we don’t know, and we’re on a journey”...I want them to have their space to do that, because that’s when the magic happens.

Parent

Another parent pointed out that interfering with physicians’ communication with other physicians could have downstream negative consequences for the patient and their family: “I wouldn’t want them to hold anything back that may help the child for fear of upsetting me or causing me alarm” (Parent).

Finally, one parent was concerned about the potential impact of Open Notes on the inpatient care workflow:

I don’t want to make their jobs more difficult as doctors, and I don’t want to burden the nurses and other medical staff with all the questions that this could bring up. I mean, I’m very sensitive to the nurses’ time. There are days when the nurses just don’t have enough hours in the day to take care of everybody they’ve got.

Parent

#### Medical Jargon

One nurse was concerned about the medical jargon present in the notes and the need to simplify the language for parent viewing based on their experience with the typical educational materials provided to patients:

[E]very teaching material that we give to patients and families goes to our learning center and gets worded to be at...the fifth-grade level or something like that. So even if there’s not medical jargon [in the note], I worry that the language is very far beyond a good portion of families’ reading abilities.

Nurse

One parent wondered rhetorically whether duplicate notetaking would now be required for 2 physicians to communicate with each other:

If you’ve got to write medical notes at the seventh-grade level, they aren’t medical notes anymore. [N]ow they’ve got to have a different system that they can put their true medical jargon in so that the next specialist knows exactly what they’re looking for.

Parent

#### Communication

The theme of communication was mentioned as a benefit in all 5 focus groups; however, it was also noted as a challenge presented by Open Notes. For example, one resident pointed to the complexity induced by the multiple readers and writers involved in note production and the resulting difficulty in interpreting what was meant:

The night team, the two residents on the night taking care of the entire hospital, if now they have to start answering questions that came out about a wording in a note...I can just imagine the increased number of nurses’ pages saying, hey, [the parents] want you to come talk about this note. And that night person isn’t the one who wrote the note. They can’t necessarily say exactly what that person meant at that point in time.

Resident

These parents also perceived that an intricate balancing act is involved when a writer represents a reader in a note:

[P]roviders noticing family dynamics and commenting on that...could turn into something quite difficult, if there’s a family dynamic that suggests an excessive amount of control or perhaps abuse...having that show up in a note that everyone is seeing...would also be a very delicate circumstance.

Parent

Another parent commented on the transparency of notes as a communication challenge:

The reason I feel nervous is it changes audience, timing, and delivery all at once. And that’s a lot.

Parent

#### Sensitive Content

Among the health care professionals, residents were most vocal about the challenge presented by sensitive content. Specific examples of potentially problematic notes included comments about the family itself:

[I]f there’s things you don’t want the family to know, like you’re considering...they’re neglecting their child, like how are you going to write that here that is friendly?

Resident

One resident described a potential negative effect on future parent readers who might be frightened by the differential diagnosis process encoded in the note:

[S]ometimes we put...malignancy in the differential diagnosis. And parents, once they read “malignancy,” they don’t care about anything else. Like once somebody hears “cancer,” like that’s the end of their mindset. So, it is going to affect our assessments because we won’t be able to be as clear or as thorough...thinking like how a parent is going to...react to this information.

Resident

One parent illustrated this phenomenon when they said the following in their focus group:

I think that there’s nothing worse than getting information and feeling like what does that mean? It sounds really ominous. You know, you see a word like “lesion” or “tumor”...and all of a sudden your creative mind runs loose. Weekends and nights are really difficult for things like that.

Parent

#### Decreasing Trust

Both residents and nurses said that allowing parents access to notes had the potential to reduce the trust parents placed on their child’s physician. A nurse gave an example of a situation in which a decision had been made to withhold certain information from the family:

I’m just thinking of a specific patient that I had recently...we were concerned about potential conversion disorder, which was a discussion that was had by the medical team but not with the family, because this was a family that was already extremely anxious and extremely...critical of everything that we were doing. And I was already getting questions about...the family being annoyed with the doctors. And obviously, you know, we stick up for our team. But...[Open Notes is] going to put us in uncomfortable, awkward positions more frequently, I guess?...[W]e already get that sometimes.

Nurse

A resident spoke about another possible effect of increased transparency on a trusting relationship—the assumption by parents that if one document was open to them, everything could be and should be transparent:

[I]f you do the precedent of...sharing some notes and not others, there’s a question of why not? I think that is going to further hurt the relationship in a negative way...why are you hiding? You’re not being forthcoming.

Resident

One parent voiced the same concern when she commented that, counterintuitively, parents might experience decreased trust in their physicians through increased transparency. She argued that they would know that their physician’s writing, the documentation of their thought process, was being changed through the expectation of that parent reader:

I would feel like I couldn’t trust my physician, because...they were filtering themselves through the hope of this new tool...I want the transparency. I want [the physicians] to go, hey, we could be wrong. And I want the doctors to always have the freedom to go “I’m thinking about this. I could be going down the wrong road.” And with this tool, no doctor is going to want to say that in a note.

Parent

## Discussion

### Principal Findings

Open Notes advocates have cited compelling reasons to open clinical documentation to patient and caregiver readership in real time. However, research on this question has focused almost exclusively on adult outpatients. This investigation included participants previously underrepresented in the Open Notes research—parents of hospitalized children. All focus groups identified many potential benefits of inpatient Open Notes. These included the enhanced sharing of information between the health care team and absent family members; increasing information for parents to review, thus adding to their knowledge base; providing parents with a sense of structure, enabling them to plan and organize; improving quality assurance for the health care system by involving parents as viewers, commenters, and potential correctors of the record; and illuminating the clinical communication process itself, thus educating and reassuring parents about the care process.

Potential challenges were also voiced. Full transparency of notes carries a risk of reflection: parents might be reading about themselves. In addition, members of all focus groups expressed concern that the process put a burden on health care providers by altering the nature of the note and the note-writing process itself. Parents were worried that these changes would have negative effects on their relationship with their child’s physician.

Several benefits discussed in the parent focus group were recurring themes in the Copying Letters research literature. One parent pointed to the capacity of notes to reinforce and recapitulate information that had already been conveyed. Partridge [15] was originally motivated to copy letters to try and solve this very problem: “Parents and patients often do not remember accurately what doctors have told them.” The parents investigated by Richards et al [34] agreed—75% of the parents saying that the letter “reminded me what was said in clinic.” Recapitulation was the most frequently mentioned benefit by the 100 parents interviewed by [17], one of whom further validated this perspective when they said:

The things in the letter are helpful, like the dosage of medicines to give. When you are there, it tends to go in one ear and out the other...when you are talking in the hospital, we were worried, so you don’t take in what’s said, so the letter helps a great deal.

Parent

Liapi et al [19] also found that their parent respondents liked the summary of the office visit because “it is difficult sometimes to absorb all that the doctor says in the clinic.”

Two challenges identified by the participants in this study echoed those voiced by physicians in several Copying Letters studies. The use and readability of medical jargon was mentioned as a potential issue that recurred in nearly all focus groups. Early Copying Letters studies also mentioned jargon as a prospective fear among clinicians who cited this as a reason not to provide copied letters. They believed this, in part, out of concern that parents would not be able to understand medical language and, in part, because avoiding jargon because of a future patient reader would require the physician to "talk down" and degrade communication with other physicians, thus affecting the content and quality of the letters [34,35]. It is interesting that when this theoretical proposition was actually tested by researchers of Copying Letters, parents who had difficulty reading the notes appeared to be in the minority. Cowper and Lenton [17] reported that 96.2% of parents found the language used in the letters “easy to understand.” The same result was found years later by Liapi [19]: not one of the 200 parents surveyed experienced an increase in anxiety upon reading their copied letter, and of the 200 parents, only 2 reported any difficulty in understanding medical terminology. Thus, a considerable gap existed between the prospective concerns expressed by clinicians and the actual parent experience.

The same was true for worries about sensitive content. A parent in this study told the other members of their focus group: “You know, you see a word like ‘lesion’ or ‘tumor’*...*and all of a sudden your creative mind runs loose.” As in the case of medical jargon, problems with content recur in the Copying Letters literature as a prospective concern among clinicians; however, like medical jargon, it appears to be a real concern only for a minority of readers. Partridge [15] reported that only 6.8% (9/133) of parents were “seriously upset” by what they read in their copied letters, either because they felt that their parenting was being criticized or because they disagreed with the content. Liapi et al [19] found only one complaint about content: out of 200 parents, 2 “felt that the letter did not accurately describe what they thought was the cause of the child’s symptoms.” Only 7.8% of the parents surveyed by Amirav et al [18] said that they felt more anxious after reading their child’s letter.

The focus groups’ ruminations on Open Notes are reminiscent of 2 specific "syndromes" of digital disruption in the wake of EHR implementation: digital deceleration and hypervigilance. Health care systems affected by digital deceleration experience reduced efficiency; digitally hypervigilant individuals are prone to anxiety and overreaction in the face of change [5]. The parents in this study expressed anxiety in remarkably similar ways to health care staff in the same hospital—to the nervous clinicians identified during the Copying Letters initiative in the United Kingdom and the primary and specialist providers surveyed by Richards et al [34]. Like health care professionals, these parents express prospective worries—they “presuffer”—about exposing clinical notes to patients’ families before any notes have actually been released. They recognize that the nature of the note itself can be changed through increased transparency and are fearful of the downstream effects of this change. Parental anxieties reveal themselves in comments about note writing: “I can’t fathom physicians needing to tone it into a different format,” as one parent puts it; another says “They aren’t medical notes anymore if you’ve got to write medical notes at the seventh-grade level.” The boundary between family and provider could potentially be violated because changing the potential readership changes the actual authorship:

If there’s a family dynamic that suggests an excessive amount of control or perhaps abuse...having that show up in a note that everyone is seeing...would...be a very delicate circumstance.

Parent

They have paid careful attention to the clinical documentation process, from the creation of notes to the reading and sharing of the notes, and acknowledged the importance of notes in the clinicians’ workflow. In so doing, these parents repeatedly assume this perspective, as they advocate for the clinical team. These findings highlight the continuing need for clear communication about documentation between parents and providers, including communication about note sharing itself.

### Limitations

This study has some limitations. Qualitative data elicited from focus groups are not intended to be generalizable but instead provide the rich context necessary to inform the development of intervention and implementation strategies, in this case, the sharing of inpatient notes. All participants were volunteers; their views may not represent the general pediatric inpatient parent population. For example, some focus group participants may have had experience with Open Notes in other clinical settings. Whether the anticipated benefits and concerns elicited from the participants in this study will translate into actual outcomes is unknown and an important area for investigation. The impact of sharing notes of other clinicians, such as nurses and physical and occupational therapists, is also a rich area for future research. The benefits, challenges, and impacts of note sharing in the case of adolescent patients are important areas for future investigation.

### Conclusions

Sociologist Marc Berg has argued that the medical record is “a force in itself, mediating the relations that act and work through it*...*The medical record achieves this role through practices of reading and writing” [36]. Until recently, the patient has not participated in medical record viewing. As Hays [37] explains:

Health care professionals have usurped the power to represent patients in the system...and the health record is the primary and most powerful means of accomplishing this...Although the (subjective) voice of the patient is heard, regarding each problem articulated by the nurse, the patient is not a full-fledged member of the fellowship of discourse, is not a reader of the chart and has no responsibility for exchange of the written text.

Thus, providing patients and, in the case of pediatrics, caregivers with access to medical records via patient portals increases the flow of information and, in turn, their ability to participate in the discourse of their care.

At the same time, we must acknowledge the transformative and potentially disruptive nature of this change in the work and dynamic of health care teams. This was a dynamic perceived by parents of pediatric patients themselves. Parents in this study demonstrated not only that they act as monitors and guardians of their children’s health but also that they are observers of the clinical processes taking place in the hospital and at their child’s bedside. This includes the clinical documentation process, from the creation of notes to the reading and sharing of the notes. Parents acknowledge not only the importance of notes in the clinicians’ workflow but also their collaboration with providers as part of the health care team.

## Abbreviations

EHR: electronic health record
